# The Renfrew Unified Treatment for Eating Disorders and Comorbidity: Long-Term Effects of an Evidence-Based Practice Implementation in Residential Treatment

**DOI:** 10.3389/fpsyt.2021.641601

**Published:** 2021-03-05

**Authors:** Heather Thompson-Brenner, Simar Singh, Taylor Gardner, Gayle E. Brooks, Melanie T. Smith, Michael R. Lowe, James F. Boswell

**Affiliations:** ^1^The Renfrew Center, Coconut Creek, FL, United States; ^2^Department of Psychology, Drexel University, Philadelphia, PA, United States; ^3^Department of Psychology, University at Albany, Albany, NY, United States

**Keywords:** eating disorder, evidence-based practice, residential treatment, implementation research, emotion intolerance, sustainability

## Abstract

**Background:** The Renfrew Unified Treatment for Eating Disorders and Comorbidity (UT) is a transdiagnostic, emotion-focused treatment adapted for use in residential group treatment. This study examined the effect of UT implementation across five years of treatment delivery.

**Methods:** Data were collected by questionnaire at admission, discharge (DC), and 6-month follow-up (6MFU). Patient outcomes were measured by the Eating Disorder Examination-Questionnaire, Center for Epidemiologic Studies-Depression Scale, Brief Experiential Avoidance Questionnaire (BEAQ), Anxiety Sensitivity Index, and Southampton Mindfulness Scale. Data were analyzed for *N* = 345 patients treated with treatment-as-usual (TAU), and *N* = 2,763 treated with the UT in subsequent years.

**Results:** Results from multilevel models demonstrated a significant interaction between implementation status (TAU vs. UT) and time, both linear and quadratic, for the depression, experiential avoidance, anxiety sensitivity, and mindfulness variables. Patients treated with the UT showed more improvement in these variables on average, as well as more rebound between DC and 6MFU. Results from multilevel models examining eating disorder outcome showed no significant difference between the TAU and UT for the full sample, but a significant three-way interaction indicated that the UT produced more improvement in the EDE-Q relative to the TAU particularly for patients who entered treatment with high levels of experiential avoidance (BEAQ score).

**Conclusion:** This long-term study of a transdiagnostic, evidence-based treatment in residential care for eating disorders and comorbidity suggests implementation was associated with beneficial effects on depression and emotion function outcomes, as well as eating disorder severity for patients with high levels of baseline emotion regulation problems. These effects did not appear to diminish in the 5 years following initial implementation.

## Introduction

Eating disorders (EDs), including anorexia nervosa (AN), bulimia nervosa (BN), binge eating disorder (BED), and “otherwise specified” eating disorders (OSFED), range widely in presentation and severity (1–3). Treatment options exist on a continuum of care, including outpatient, intensive outpatient, partial hospital, and residential treatment, with residential treatment recommended for individuals with severe, complex, and treatment-resistant symptoms (3, 4).

The number of private residential programs in the United States has increased in recent years; (5–8) however, outcome data regarding evidence-based practices (EBPs) in residential treatment for EDs remain scarce (6, 7, 9, 10). A recent review located only *N* = 19 discrete studies of any residential treatment outcomes (10). Among the noted limitations, most studies lacked controls and less than half included follow-up data; when reported, follow-up response rates were low (5, 10). No randomized, controlled comparisons of manualized residential treatments have been reported (10).

There are many obstacles to full implementation and controlled research for EBPs in residential ED programs. Patients in intensive settings typically struggle with two or more co-morbid psychiatric disorders (11), and residential treatment providers suggest that existing manuals for EDs do not adequately address comorbidity (12). In addition, residential programs provide individual and group therapy many times throughout the week, yet EBPs are typically designed to be delivered once or twice per week and lack guidance for adaptation. Furthermore, residential programs provide intensive structural regulation and staff oversight to eliminate ED behaviors such as restriction, binge eating, and purging, while behavioral regulation is a primary focus of most manualized treatments (13, 14).

In addition to interventions that directly address ED behaviors and cognitions, investigators have highlighted the possible importance of emotion regulation as a treatment target in psychotherapy for EDs (15). One recent review concluded that both AN and BN had demonstrated consistent associations with particular emotion regulation difficulties, including lack of awareness of emotions, lack of acceptance of emotions, negative beliefs about emotions or coping, and avoidance/suppression of emotions. While EPBs for EDs that address both ED symptoms and emotion regulation have demonstrated benefits for individuals with EDs (14, 16, 17), additional research is needed to establish whether emotion regulation interventions demonstrate significantly better outcomes than other interventions for EDs, and/or benefits are observed particularly for individuals with higher levels of emotion regulation problems.

Our research group conducted one preliminary study of an integrative EBP for EDs and transdiagnostic emotion functioning in residential care, which compared outcomes from patients who were treated in the first year following implementation to patients who received treatment-as-usual (TAU) prior to implementation (17). The multi-modal, evidence-based residential treatment, adapted from the Unified Protocol developed by Barlow and colleagues (18), is now known as the Renfrew Unified Treatment for Eating Disorders and Comorbidity (19, 20), or Unified Treatment (UT). The UT is a manualized, transdiagnostic approach, with structured groups that address EDs and comorbid disorders using integrative emotion-focused cognitive, behavioral, and experiential interventions. UT modules and research to support their use with EDs are presented in Table 1. In that preliminary study, analyses indicated that patients treated with the UT showed more improvement in dimensions of psychopathology directly addressed in the UT manual as putative mechanisms—experiential avoidance, anxiety sensitivity, and mindfulness—relative to patients in TAU (17). Treatment effects for ED and depression treatment outcomes were large in both UT and TAU groups, and did not differ by group (17).

**Table 1:** Unified treatment common elements, techniques, and eating disorder research examples.

**Common elements**	**Techniques**	**Basic Supporting Research (Examples)**	**Treatment research (Examples)**
Motivation enhancement	Identification of “pros” and “cons” of change; identification of goals and immediate steps for change	Individuals with EDs show low motivation to change; motivation and readiness predicts outcome in EDs (21)	Motivational Interviewing increases motivation in EDs (21) and is part of CBT-E, (13) ICAT. (14)
Function of emotions	Understanding of adaptive functions of emotions; 3-component model (thoughts, behaviors, sensations); antecedents, responses, and consequences of emotions	Individuals with EDs lack emotion awareness and show negative beliefs about emotion (22, 23). Negative affect is an ED risk factor (24–26)	Mindfulness exercises benefit patients with EDs and are included in ICAT, CBT-E, DBT, (27) EABT, (16) and ACT for EDs (28)
Emotion awareness training	Development of nonjudgmental, present-focused awareness	EDs are associated with lack of emotion awareness, lack of emotion acceptance, negative beliefs about emotion, poor mindfulness and high emotion non-acceptance (29–31)	Mindfulness exercises show benefit for patients with EDs (32, 33) and related components are included in DBT, ICAT, EABT, and ACT
Cognitive appraisal & reappraisal	Identification of subjectivity and emotional influence on cognition; probability over-estimation and catastrophizing; core negative appraisals (downward arrow technique)	Negative cognitions such as thin-ideal internalization are associated with the development and maintenance of behavioral EDs (23, 26, 34); negative cognitions associated with food, eating, perfectionism, exercise, body image, are components of eating disorders	Cognitive therapy shows benefit for shape and weight concerns, (35, 36) and related components are included in CBT-E, EABT, and ACT
Avoidance and emotion-driven behaviors	Identification of maladaptive emotion avoidance and emotion-driven behaviors; promotion of adaptive alternatives	EDs are characterized by avoidance of emotion, (23, 31, 32) as well as checking (37, 38) and other rituals (39)	Related interventions or components are included in DBT, EABT, ICAT, ACT, and IPT (40)
Interoceptive awareness & tolerance	Engagement in exercises which evoke physical sensations similar to those of strong emotions (i.e., interoceptive exposure)	EDs are associated with low interoceptive awareness (41–46)	Interoceptive practices, such as appetite awareness training, have shown benefit for individuals with EDs (47, 48)
Emotion exposures	Construction of a hierarchy of avoided and distressing situations; planning and engagement in exposures	EDs are characterized by avoidance of emotion (31, 49), avoidance of viewing or revealing the body (38, 50) and avoidance of feared foods (13)	Related components are included in CBT-E (e.g., weighing and introduction of feared foods), EABT & ACT, and AN-EXRP (51–53)

*CBT-E, cognitive behavior therapy-enhanced; ICAT, integrative cognitive-affective therapy; DBT, dialectical behavior therapy; EABT, emotion acceptance behavior therapy; IPT, interpersonal psychotherapy. Elements of this table are included in Thompson-Brenner et al. (20)*.

EBP implementation research has increasingly focused on “sustainability.” Compared to the step-wise changes that characterize the early stages of implementation, (e.g., adoption, initial implementation) (54, 55), sustainability can be defined as the consistent usage of key program components demonstrating continued achievement of intended outcomes over an extended period of time (56, 57). Extensive implementation research suggests that even when the challenges of implementing EBPs with fidelity have been surmounted, it is difficult to maintain consistent use, as well as intended effects, over longer periods of time (56).

This community case-report focuses on the sustainability of the effects of the Renfrew UT in residential ED care across two sites, over 6 years of observation. This study aimed to investigate whether: (1) significant differences in effect of the UT and TAU at discharge and 6-month follow-up were observed across 5 years of treatment delivery, (2) there were specific effects of the UT relative to TAU for individuals with higher levels of emotional intolerance, and (3) the large treatment effect sizes for outcomes that were observed one-year post-implementation were still observed multiple years after the initial implementation.

## Methods and Materials

### Treatment Approach and Implementation Process

Patient-participants were in residence and received treatment between admission and discharge (DC). Daily therapeutic interventions included: structured daily activities; dietitian-prescribed and staff-supervised meals and snacks; 3–4 therapeutic group sessions per day; and individual meetings with psychotherapists, dietitians and psychiatrists. The frequency and intensity of all types of treatment (e.g., group therapy, individual therapy) and discipline (e.g., psychotherapy, psychiatry, nutrition) remained consistent across time.

The UP was selected for adaptation and implementation based on many considerations. In residential treatment, food intake and behavioral symptoms are regulated, limiting the application of several common outpatient empirically-supported treatments for EDs (e.g., CBT and FBT). Common manualized treatments for EDs do not fully address common and severe comorbidities, including social anxiety disorder, obsessive-compulsive disorder, and post-traumatic stress disorder. Previoiusly, residential treatment programs had incorporated elements of different empirically-supported treatments for EDs (e.g., DBT and ACT groups), but in an eclectic rather than integrated fashion, (10) which the Renfrew team felt was difficult to unify across sites and levels of care.

Training provided prior to implementation, as well as to all new employees, is program-wide and mandatory for all members of the clinical staff across disciplines at both sites. At the time of implementation, the training department conducted on-site three-day didactic and experiential training in the UT that was based on the training provided to clinical leadership by UP trainers. New staff from all clinical disciplines participate in onboarding training during their first weeks of employment, consisting of 8 hours of interactive web-based training with trainers certified in the UP. The training provides in-depth exploration and application of the theoretical principles in the UT, including experiential exercises based on the exercises completed during UT groups. Additional discipline-specific training (e.g., therapy, nutrition, psychiatry/medical/nursing) more specifically focuses on application of UT principles and interventions in various roles. Staff performance is continuously monitored following training through the review of audio-recorded group therapy sessions by supervisors and trainers resulting in substantive feedback and coaching. [See Table 2 for a brief comparison of UT and TAU treatment and training; see Thompson-Brenner et al. (20), (17) for in-depth description of the UT emotion-focused approach including the adaptation and implementation processes].

**Table 2:** Comparison of unified treatment and treatment-as usual.

	**Unified Treatment**	**Treatment-As-Usual**
Treatment: frequency and intensity	Daytime, Overnight, and Weekend Milieu Supervision	Yes
	5-6 Group Therapy sessions per day	Yes
	3 Individual Therapy sessions per week	Yes
	1 Family Therapy session per week	Yes
	Nutrition & Psychiatry Counseling, Nursing checks	Yes
Treatment: content	Manualized Unified Treatment; adapted from Unified Protocol for ED use in residential programs, structured interventions for motivation, emotion awareness and acceptance, cognition and behavior change	Eclectic and idiosyncratic, developed by practitioners and approved by program, informed by principles of feminist-relational theory
	Based on evidence-based common elements	Some *ad hoc* Acceptance and Commitment Therapy and Cognitive Reprocessing Therapy
	Daily and weekly structured symptom monitoring	No
Training: frequency and intensity	Weekly on-site supervision of practitioners	Yes
	Centralized supervision of supervisors	No
	Manualized Fidelity Ratings	No
	Yearly Clinical Retreat	Yes
	Introductory 8 h of training and supervision on treatment model	No
	Unified manuals and materials for disciplines and treatment types	Eclectic guidance and structure for disciplines and treatment types
Training: content	Training in the Unified Treatment	Eclectic topical training

The implementation date at each residential site was the date at which the clinical staff completed intensive training, adopted the manual, and provided UT supervision. Fidelity to the UT protocol was assessed by external raters, across sites and groups, in the year following implementation; fidelity was established to be adequate (17). In subsequent years between the initial implementation period and the end of data collection in this report, training, supervision and manual materials were assessed and adjusted in an iterative process, and components of the multi-modal treatments (e.g., nutrition counseling, family therapy, expressive therapies) were adjusted to improve congruence with the UT.

### Patient Assessments and Procedures

The study period ran from 2014 through 5 years post-implementation. Admission data for the TAU group were collected from February 2014 until implementation date (October 2014 at one site, and March 2015 at the other). Admission data from the UT group were collected from implementation date until November 2019. Throughout the study period, residential patient-participants completed standard admission procedures, including screening and psychiatric interview assessment of EDs, co-occurring diagnoses, and medical/behavioral stability. All routinely presenting patients completed a standard battery of computerized self-report assessments for internal outcome monitoring purposes at admission and DC. Patients who had completed at least one survey were contacted via email and provided a secure web link for remote completion of the 6MFU assessment. Patients received $30 Amazon gift cards for completion of 6MFU. All research activities were approved by institutional review boards at The Renfrew Center and Drexel University.

Only patients consenting to have their data used for research (*N* = 3775; 95.2% of all patients) were considered for inclusion in the present study. Exclusions included: (1) previous admission during the data collection period (*n* = 509); (2) length of stay <7 days (*n* = 117); (3) admission date past the fifth year of implementation (*n* = 41). These exclusions yielded a final sample size of 3,108 eligible for analyses (TAU: *n* = 345; UT: *n* = 2,763) across two residential treatment sites.

### ED Symptom Severity

The Eating Disorder Examination-Questionnaire (EDE-Q) (58) is a 28-item self-report measure. The global score was used to examine overall eating disorder severity, ranging from 0 to 6 with higher scores indicating more severe eating disorder symptoms (sample α= 0.87).

### Depressive Symptoms

The Center for Epidemiologic Studies Depression Scale (CES-D) (59) is a 20-item self-report assessment. Items on a Likert scale range from 0 (rarely or none of the time) to 3 (most of all of the time). The total score ranges from 0 to 60 and higher scores indicate more symptomology (sample α= 0.88).

### Experiential Avoidance

The Brief Experiential Avoidance Questionnaire (BEAQ) (60) is a 15-item self-report measure. The individual items closely match dimensions of emotion regulation problems observed to be elevated in EDs, such as lack of emotional awareness (e.g., “It's hard for me to know what I am feeling”); lack of emotion acceptance (e.g., “One of my big goals is to be free from painful emotions”); emotion avoidance (e.g., “I rarely do something if there is a chance that it will upset me”); emotion suppression (e.g., When unpleasant memories come to me, I try to put them out of my mind”); and negative beliefs about emotion (e.g., “The key to a good life is never feeling any pain”). Items are on a Likert scale from 1 (strongly disagree) to 6 (strongly agree). Scores can range from 15 to 60, with higher scores indicating more experiential avoidance (sample α= 0.84). In prior research, the 62-item Multidimensional Experiential Avoidance Questionnaire (MEAQ) (61) was utilized; however, the brief version was highly correlated with the longer version and reduced participant burden. Participants who had completed the MEAQ had their scores re-coded using the 15 items of the BEAQ.

### Anxiety Sensitivity

The Anxiety Sensitivity Index (ASI) (62) is a 16 item self-report measure that assesses negative attitudes toward the physical sensations of anxiety (e.g., “It scares me when my heart beats rapidly”). Items are on a Likert Scale from 0 (very little) to 4 (very much). The total score ranges from 0 to 64, with higher scores indicating more anxiety sensitivity (sample α= 0.87).

### Mindfulness

The Southampton Mindfulness Questionnaire (SMQ) (63) is a 16-item self-report measure of the goals of mindfulness training, including acceptance of emotion (e.g., “Usually when I experience distressing thoughts and images, I try to just experience the thoughts or images without judging them”) and particular observations about emotion (e.g., “Usually when I experience distressing thoughts and images, I notice how brief the thoughts and images really are”). Items are on a Likert scale from 0 (disagree totally) to 6 (agree totally). The total score ranges from 0 to 96, with higher scores indicating more mindfulness (sample α= 0.87).

### Patient Diagnoses

Primary diagnoses were established via a two-step procedure. Trained assessors conducted intake interviews over the phone prior to admission, which included structured assessment of each diagnostic criterion for ED diagnosis. Co-occurring symptoms were assessed in the intake interview. Following admission, the ED and co-occurring diagnoses were confirmed by a semi-structured psychiatric interview administered by a psychiatrist. BMI was assessed at intake and DC using electronic medical scales. When new diagnostic criteria were added in DSM-V (e.g., for ARFID and OSFED), the rates of particular diagnoses changed in accordance with the new criteria.

### Fidelity Monitoring

Ongoing supervision and monitoring was used to maintain fidelity to the UT. UT groups are digitally recorded and uploaded to an internal, secure server. Site supervisors, with established fidelity to the UT method, randomly select one recording per week from each supervisee's recordings, listen to the entire recording, and complete the fidelity measure, giving a score from 0–100% adherence based on the presence or absence of required group content. The supervisor then uses the adherence rating, as well as observations about clinician skills (e.g., group engagement and cohesiveness, warmth, empathy and understanding), to provide overall ratings of adherence and quality. This assessment forms the basis of targeted, substantive feedback in supervision. Additionally, each week one of the supervisor-reviewed recordings is rated by a member of the Training Department. The ratings and feedback of the trainer and supervisor are compared and discussed in a weekly “supervision of supervision” session and the training department uses information from ongoing review of group recordings to inform training initiatives for the organization to maintain fidelity of the UT.

External researchers rated a limited set of fidelity ratings for a separate study in 2019 (31). Observer-rated adherence was in the excellent range, with scores ranging from 80–100% across all rated sessions (*M* = 96.99%, *SD* = 0.07). Observer-rated quality and competence were also good, with all individual item means for adherence items falling within the “high quality” to “very high quality” range and all individual item means for the competence items falling within the “good” to “excellent” range (64).

### Statistical Plan

#### Effect of UT Implementation

Multilevel models were used to analyze whether change in outcome over the course of treatment and follow-up (i.e., admission to DC to 6MFU) varied as a result of UT implementation. Growth curves were modeled with second-order orthogonal polynomials and fixed effects of UT status on all time terms. The Pre-UT condition was treated as the baseline, and parameters were estimated for the Post-UT condition. Time was modeled continuously in units of 6-months (i.e., dividing number of days by 183, the number of days in 6-months), with each participant's admission coded as 0, to promote convergence across all models. Comparison of model fit using ANOVA revealed that a random effects structure allowing variation in each participant's baseline score and quadratic trajectory over time resulted in best model fit, across all outcomes. The fixed effects of time (linear and quadratic) and their interaction with UT status were added sequentially, and effects on model fit were also evaluated using ANOVA. Finally, the moderating effect of baseline experiential avoidance (i.e., BEAQ admission scores) on UT status in relation to outcome was investigated in an exploratory manner. BEAQ score was of particular interest because the treatment approach, which focused on awareness of emotion and the reduction of emotion avoidance, might be hypothesized to be of particular relative benefit to individuals with higher levels of emotional avoidance at baseline. Additionally, because the UT and TAU groups significantly differed in their number of comorbidities and frequency of AN-R diagnoses at baseline, these variables were included as time-varying (linear and quadratic) covariates. All multilevel analyses were carried out in RStudio version 1.2, (65) using the lme4 and lmer packages.

#### Implementation Sustainment

Effect sizes (Cohen's *d*) for calculated separately for subsamples of patients who were admitted in each calendar year across the study time period. All sustainment analyses were conducted in SPSS, v.27 (66).

## Results

### Response Rates

Response rates across timepoints and years of implementation are shown in Table 3. Rates of completion at admission were consistently high, while response rates for DC and 6MFU varied across years. To assess for response bias, chi-square tests were used to examine response rates across the TAU and UT groups, and ANOVAs were used to examine whether (1) TAU v. UT and (2) 6MFU completers v. non-completers, significantly differed on scores at admission.

**Table 3:** Patient characteristics.

**Demographics**	**Pre-UT**	**1 year post**	**2 years post**	**3 years post**	**4 years post**	**5 years post**
	***n* = 345**	***n* = 491**	***n* = 587**	***n* = 563**	***n* = 604**	***n* = 518**
**Ethnicity (%)**						
White	78.3	79	82.8	80.1	79.6	80.1
Hispanic	5.8	4.9	5.5	6.6	7.1	6.4
African-American	1.2	1.8	1.4	2.1	2.2	3.1
Asian/Pacific Islander	1.4	2.6	2.4	2.1	2	2.5
Multiracial	2.6	2.4	3.4	4	3.8	3.9
Other	2	1.4	2.4	2.5	2.8	1.9
Declined to respond	8.7	7.3	1.9	1.8	1.8	1.7
**Age**						
Range	14–63	13–66	13–69	13–75	14–65	14–73
M ± SD	26.3 ± 11.0	24.5 ± 13.0	25.8 ± 11.3	25.1 ± 10.9	24.2 ± 10.2	26.2 ± 12.3
Adolescents (%)	20.6	23	22	23.6	26.5	22.4
Adults (%)	79.4	77	78	76.4	73.5	77.6
**LOS (M** **±** **SD)**	32 ±14	30 ± 13	32 ± 13	33 ± 16	33 ± 15	32 ± 13
**ED diagnosis** (%)						
AN-R	25.8	26.3	26.8	19.7	23.8	23.6
AN-BP	11	16.5	14.3	21.8	21.4	22.2
BN	31.6	31.4	28.3	29.8	24.7	23
BED	2.3	5.9	5.3	4.8	4.5	6.9
EDNOS	29	1.8	–	–	–	–
OSFED	0.3	15.3	22.7	21.3	24.2	21.6
ARFID	–	1.6	1.2	1.1	1.5	2.5
UFED	–	1.2	2.1	1.4	–	0.2
**Comorbidity (M** **±** **SD)**	2 ± 1	2 ± 1	2 ± 1	2 ± 1	2 ± 1	2 ± 1
**Site (%)**						
Northeastern US	64.9	70.5	61.5	67.9	68	63.3
Southeastern US	35.1	29.5	38.5	32.1	32	36.7
**Admission BMI (M** **±** **SD)**	22.2 ± 9.3	21.8 ± 7.6	22.4 ± 7.9	23.0 ± 9.3	22.4 ± 7.8	23.0 ± 9.0
**Baseline scores (M** **±** **SD)**						
EDE-Q	4.1 ± 1.4	4.0 ± 1.5	4.0 ± 1.5	4.0 ± 1.4	4.0 ± 1.4	4.1 ± 1.4
CES-D	36.7 ± 12.3	37.3 ± 12.5	37.4 ± 12.3	38.7 ± 11.8	37.5 ± 11.3	37.9 ± 11.8
BEAQ	57.3 ± 12.9	57.7 ± 12.3	57.8 ± 13.5	59.6 ± 13.4	59.2 ± 12.0	59.5 ± 13.3
ASI	31.6 ± 12.5	31.8 ± 12.5	33.1 ± 13.9	32.0 ± 13.2	31.8 ± 13.0	31.9 ± 12.9
SMQ	31.4 ± 16.5	34.1 ± 16.7	32.6 ± 17.1	30.0 ± 17.2	29.2 ± 16.0	30.8 ± 16.3
**Response rates (%)**						
Completed ADM	97.7	99.8	98.6	99.3	99.5	98.5
Completed DC	96.8	99.4	88.9	88.1	84.6	84.4
Completed 6MFU	42	47.3	59.8	65	68.2	61.6

*LOS, length of stay; ED, eating disorder; AN-R, anorexia nervosa-restricting subtype; AN-BP, anorexia nervosa-binge eating/purging subtype; BN, bulimia nervosa; BED, binge eating disorder; EDNOS, eating disorder not otherwise specified; OSFED, other specified feeding and eating disorder; ARFID, avoidant/restrictive food intake disorder; UNFED, unspecified feeding or eating disorder; ADM, admission; DC, discharge; 6MFU, six month follow-up*.

Chi-square analyses revealed different response rates for the TAU and UT groups, at admission, discharge, and follow-up. Response rates were significantly higher in the UT phase at admission (UT: *n* = 2,739, 99.1%; TAU: *n* = 337, 97.7%) and 6MFU (UT: *n* = 1,680, 60.8%; TAU: *n* = 145, 42.0%); however, the TAU phase demonstrated higher response rates at discharge (UT: *n* = 2,453, 88.8%; TAU: *n* = 334, 96.8%). Chi-square analyses further revealed that response rates differed in subsequent years of UT implementation (all *p*'s>0.05). Therefore, we conducted analyses to examine whether any differences were observed between responders and non-responders at baseline. No differences on any outcome measures at admission were observed comparing either discharge completers vs. non-completers or 6MFU completers v. non-completers (all *p*'s>0.05).

### Patient Demographics

Patient characteristics are reported in Table 3. Age ranged from 13 to 75 years (*M* = 25; *SD* = 0.19). The majority of the sample was White (*n* = 2492; 80.1%), and the most common ED diagnosis was bulimia nervosa (*n* = 865; 27.7%). The majority of the sample was diagnosed with at least one comorbid disorder (*n* = 2,909; 93.5%), and the most common comorbidities were mood disorders (*n* = 2507; 80.7%) and anxiety disorders (*n* = 996; 32.0%).

### Multilevel Model Results

**Figure 1:**
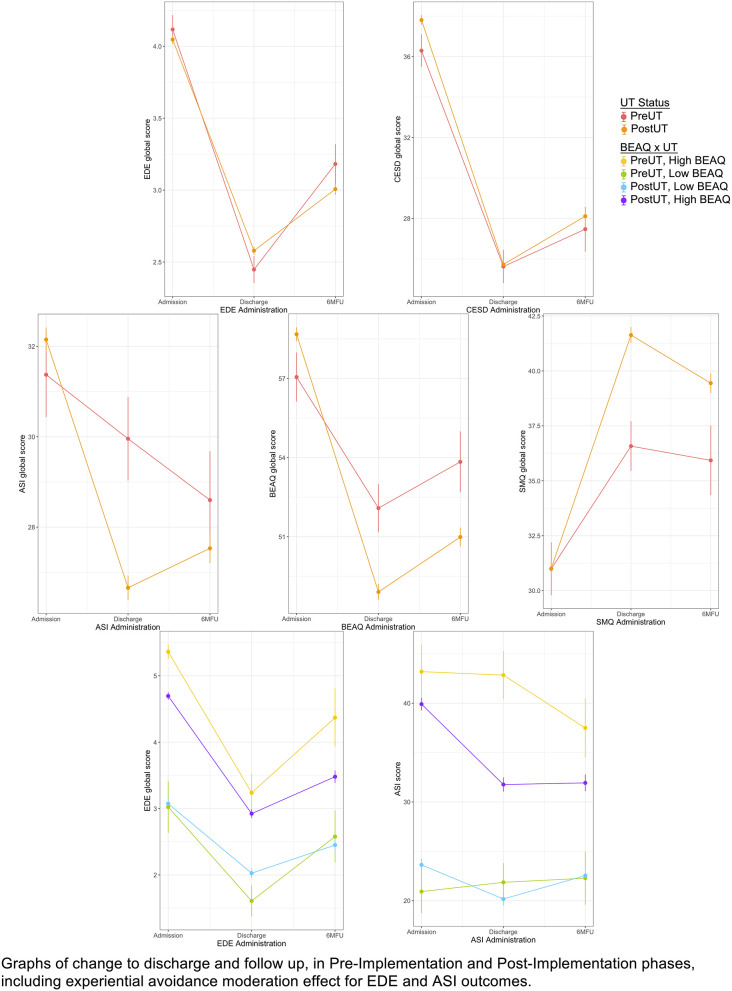
Change in outcome variables discharge to 6MFU in pre-implementation vs. post-implementation. Graphs of change to discharge and follow up, in pre-implementation and post-implementation phases, including experiential avoidance moderation effect for EDE-Q and ASI Outcomes.

Model parameters for growth curve analyses investigating the UT's effect on outcome are summarized in Table 4. All models included [1 + time (2) | ID] as the random effects structure. In determining the best fitting model for EDE-Q, the inclusion of a fixed (UT × linear time) interaction term did not improve model fit; therefore, it was dropped from the model. Similarly, the inclusion of a fixed (quadratic time) main effect did not improve model fit for the ASI; therefore, it was dropped from the model. Differences in baseline CES-D scores across the UT and TAU groups were controlled for by including a main effect of the UT (i.e. a variable reflecting differences in baseline scores in the TAU group relative to the UT group). The UT and TAU groups demonstrated comparable scores at baseline on all other outcomes; therefore, the UT main effect was not included in these models. Outcomes from multilevel models are presented in Table 4; graphs of change in outcome variables at DC and 6MFU in pre-implementation vs. post-implementation are presented in Figure 1.

**Table 4:**
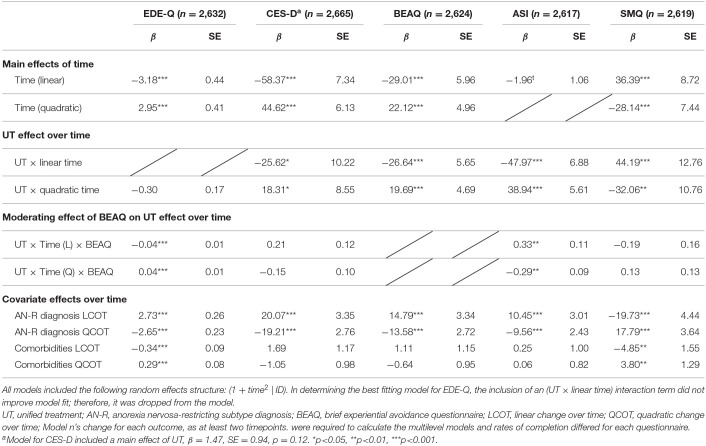
Multilevel models examining the effect of UT status on outcomes, from admission to follow-up.

#### EDE-Q

Significant linear [β = −3.18, *t*(4,324.45) = −7.30, *p* < 0.001] and quadratic [β = 2.95, *t*(4,672.92) = 7.26, *p* < 0.001] effects of time on EDE-Q were observed. The negative linear time effect indicates a decrease in EDE-Q global scores over time on average regardless of UT status, while the positive quadratic time effect indicates a general rebound in EDE scores over time, regardless of UT status. There was no significant interaction between UT status and quadratic time. As noted, the interaction between UT status and the linear time component did not improve model fit, and was not included. These results indicate that the UT was not associated with more improvements in EDE-Q relative to the TAU group.

In three-way interactions, baseline BEAQ scores showed a significant moderating effect on the relationship between UT implementation and EDE-Q linear change over time [β = −0.04, *t*(4,520.62) = 6.22, *p* < 0.001], and quadratic change over time [β = 0.04, *t*(4,867.61) = 6.02, *p* < 0.001], indicating that for individuals with higher baseline BEAQ scores, the implementation of the UT was associated with a greater decrease in EDE-Q scores relative to TAU at discharge, and lesser rebound in EDE-Q scores relative to TAU between discharge and follow-up. This suggests that the UT implementation showed a greater positive effect on EDE-Q scores relative to TAU specifically for those individuals with higher experiential avoidance scores at baseline (see Figure 1).

The covariate reflecting a baseline diagnosis of AN-R showed a positive relationship to linear change over time in EDE-Q score [β = 2.73, *t*(4,464.95) = 10.71, *p* < 0.001], and a negative relationship to quadratic change over time [β = −2.65, *t*(4,631.23) = −11.38, *p* < 0.001], indicating those participants with AN-R showed less steep EDE-Q change from admission to discharge, and less rebound between discharge and follow-up relative to the group with other ED diagnoses. The covariate reflecting the number of co-occurring diagnoses at baseline showed significant negative relationship with linear change over time [β = −0.34, *t*(4,426.19) = −3.68, *p* < 0.001], and significant positive quadratic change over time [β = 0.29, *t*(4,577.81) = 3.49, *p* < 0.001], indicating that the presence of more comorbid diagnoses predicted a steeper change in EDE-Q score by discharge, and steeper rebound between discharge and follow-up.

#### CESD

Significant linear [β = −58.37, *t*(3,479.79) = −7.96, *p* < 0.001] and quadratic [β = 44.62, *t*(3,723.18) = 7.28, *p* < 0.001] effects of time on CESD outcome were observed. The negative linear time effect indicates an average decrease in CESD scores over time while the positive quadratic time effect indicates a general rebound in CESD scores over time, regardless of UT status. As noted in Table 4, the main effect of UT status on CESD was included to control for differences in baseline scores between the UT and TAU groups, but this effect was not significant at the *p* < 0.05 level in the final model. UT status showed significant interactions with linear change over time [β = −25.62, *t*(4,321.03) = −2.51, *p* = 0.01] and quadratic change over time [β = 18.31, *t*(4,420.07) = 2.14, *p* = 0.03] suggesting that the implementation of the UT was associated with larger decrease in CESD scores overall, as well as larger rebound in CESD scores between discharge and follow-up.

In three-way interactions, baseline BEAQ did not significantly moderate the relationship between UT status and linear or quadratic change over time. The covariate reflecting a baseline diagnosis of AN-R showed a positive relationship to linear change over time [β = 20.07, *t*(5,323.75) = 6.00, *p* < 0.001], and a negative relationship to quadratic change over time [β = −19.21, *t*(4,886.84) = −6.95, *p* < 0.001], indicating those participants with AN-R showed lesser CES-D change from admission to discharge, and lesser rebound between discharge and follow-up relative to the group with other ED diagnoses. The covariate reflecting the number of co-occurring diagnoses at baseline did not show significant relationships to linear or quadratic change in CES-D scores over time.

#### BEAQ

Significant linear [β = −29.01, *t*(5,600.38) = −4.87, *p* < 0.001] and quadratic [β = 22.12, *t*(5,150.61) = 4.46, *p* < 0.001] effects of time on BEAQ outcome were observed. The negative linear time effect indicates an average decrease in BEAQ scores over time, while the positive quadratic time effect indicates a general rebound in BEAQ scores over time, regardless of UT status. There were significant interactions between UT status and both the linear [β = −26.64, *t*(5,749.05) = −4.72, *p* < 0.001] and quadratic time components [β = 19.69, *t*(5,066.31) = 4.20, *p* < 0.001]. The negative linear interaction indicates a steeper decrease in BEAQ scores over time for the UT group, compared to TAU; while the positive quadratic interaction indicates greater rebound in BEAQ scores over time in UT group compared to TAU.

The covariate reflecting a baseline diagnosis of AN-R showed a positive relationship to linear change in BEAQ score over time [β = 14.79, *t*(5,545.31) = 4.42, *p* < 0.001], and a negative relationship to quadratic change over time [β = −13.58, *t*(4,942.91) = −4.99, *p* < 0.001], indicating those participants with AN-R showed less steep BEAQ change from admission to discharge, and less rebound between discharge and follow-up relative to the group with other ED diagnoses. The covariate reflecting the number of co-occurring diagnoses at baseline did not show significant relationships to change in BEAQ scores over time.

#### ASI

The main effect of time on ASI trajectories was significant only at the trend level (*p* = 0.06; see Figure 1). There were, however, different patterns of change over time relative to UT status. UT status demonstrated a significant interactions with both the linear time component [β = −47.97, *t*(5,104.04) = −6.98, *p* < 0.001] and the quadratic time component [β = 38.94, *t*(4,626.93) = 6.94, *p* < 0.001], indicating that there was steeper decrease in ASI scores over time for the UT group compared to TAU, as well as a greater rebound in ASI scores over time, compared to those in the TAU group.

In three-way interactions, baseline BEAQ score was a significant moderator of the linear relationship between UT status and ASI scores [β = 0.33, *t*(5,127.63) = 3.05, *p* = 0.002] and also the quadratic relationship between UT status and ASI score [β = −0.29, *t*(4,476.39) = −3.18, *p* = 0.002] indicating that individuals with higher BEAQ scores at admission, relative to those with lower BEAQ scores, showed greater overall comparative improvement in ASI scores in UT group relative to TAU group. As shown in Figure 1, individuals with higher BEAQ scores in the UT showed greater improvement in ASI scores by discharge, and virtually no rebound, compared to those with high BEAQ scores in the TAU, who showed only a moderate decline in ASI scores by discharge, and additional decline in ASI scores by 6MFU.

The covariate reflecting a baseline diagnosis of AN-R showed a positive relationship to linear change in ASI score over time [β = 10.45, *t*(5,177.20) = 3.47, *p* < 0.001], and a negative relationship to quadratic change over time [β = −9.56, *t*(4,555.24) = −3.93, *p* < 0.001], indicating those participants with AN-R showed more ASI change from admission to discharge, and more rebound between discharge and follow-up relative to the group with other ED diagnoses. The covariate reflecting the number of co-occurring diagnoses at baseline did not show significant relationships to change in ASI scores over time.

#### SMQ

^1^Significant linear [β = 36.39, *t*(4,955.47) = 4.17, *p* < 0.001] and quadratic [β = −28.14, *t*(4,919.48) = −3.79, *p* < 0.01] effects of time on SMQ outcome were observed. The positive linear time effect indicates an average increase (i.e., improvement) in mindfulness scores over time, while the negative quadratic time effect indicates a general rebound in mindfulness scores over time, regardless of UT status. Results also indicated significant interactions between UT status and both the linear [β = 44.19, *t*(5,322.19) = 3.46, *p* < 0.001] and quadratic time components [β = −32.06, *t*(4,973.79) = −2.98, *p* = 0.003]. The positive linear interaction indicates a steeper increase (i.e., improvement) in SMQ scores over time for the UT group compared to TAU, while the negative quadratic interaction indicates greater rebound in SMQ scores over time in UT group compared to TAU.

In three-way interactions, baseline BEAQ did not moderate the effect of the UT implementation on SMQ scores. The covariate reflecting a baseline diagnosis of AN-R showed a negative relationship to linear change over time in SMQ score [β = −19.73, *t*(5,400.16) = −4.44, *p* < 0.001], and a positive relationship to quadratic change over time [β = 17.79, *t*(4,774.16) = 4.89, *p* < 0.001], indicating those participants with AN-R showed less SMQ change from admission to discharge, and less rebound between discharge and follow-up relative to the group with other ED diagnoses. The covariate reflecting the number of co-occurring diagnoses at baseline showed a significant negative relationship with linear change over time [β = −4.85, *t*(5,430.71) = −3.12, *p* = 0.002], and significant positive quadratic change over time [β = 3.80, *t*(4,812.25) = 2.94, *p* = 0.003], indicating that a greater number of comorbid diagnoses was associated with a slower rate of improvement in mindfulness scores from admission to follow-up, and a significant decelerating (negative) trajectory from discharge to follow-up.

### Sustainment

**Figure 2:**
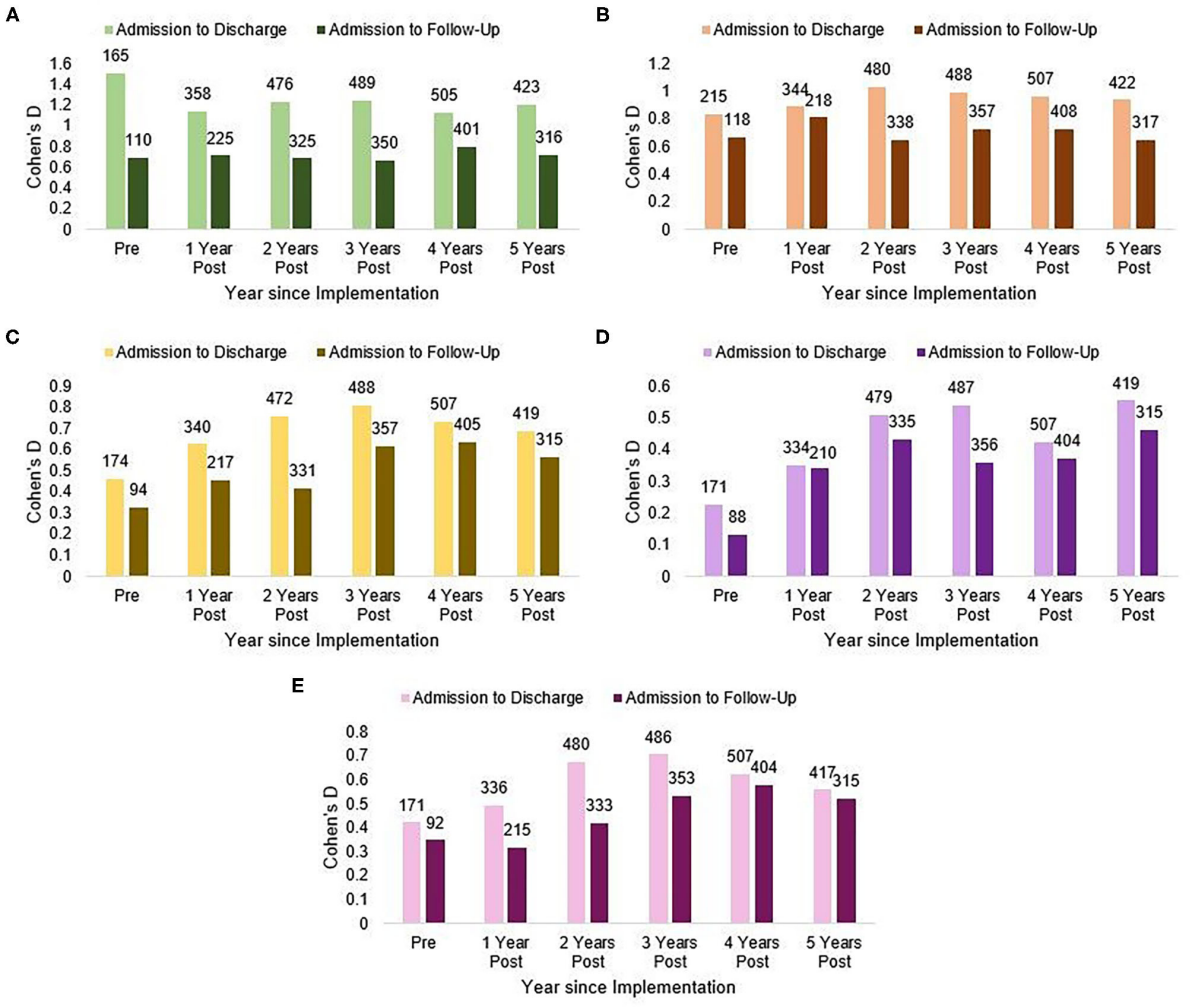
Sustainment of implementation effect over 5 years. **(A)** EDE-Q, **(B)** CES-D, **(C)** BEAQ, **(D)** ASI, and **(E)** SMQ. Number above each bar indicate the total n. EDE-Q, Eatting Disorder Examination-Questionnaire; CES-D, Center for Epidemiologic Studies Depression Scale; BEAQ, Brief Experiential Avoidance Scale; ASI, Anxiety Sensitivity Index; SMQ, Southampton Mindfulness Questionnaire.

Figure 2 shows graphs of effect sizes on each outcome variable at DC and 6MFU, across each year of data collection. Visual inspection of observed benefits for most outcome variables were maintained over time, particularly between DC and 6MFU in the first year following implementation of the UT relative to (a) TAU, and (b) subsequent years of the implementation (years 2–5). In several graphs, the improvement effect continues to increase in additional early years of implementation, and returns to slightly more moderate levels in the last two years.

## Discussion

This study investigated the effect of the implementation of the Renfrew Unified Treatment for Eating Disorders and Comorbidity (UT) (19) across a multidimensional residential treatment program at two sites in the United States, between 2014 and 2019. Overall, results demonstrate support for the effectiveness of the UT, for patients at discharge and 6-month follow-up, that was not diminished across multiple years following the initial implementation effort.

Analyses of the ED-specific outcome did not detect a significant difference in effect for individuals treated in the pre-implementation TAU phase and those treated with the UT over 5 years of data collection. These results were similar to those reported in a prior report that examined 1 year of post-implementation data (17). However, in three-way interactions, the baseline level of experiential avoidance moderated the relationship between implementation and EDE-Q change: individuals with higher baseline BEAQ scores showed a greater decrease in EDE-Q score over time in the UT relative to the TAU, whereas those with lower baseline BEAQ scores did not show this relative benefit for the UT on EDE-Q score. This finding suggests that the UT shows relative benefit in ED symptoms for those patients who have one form of emotional dysregulation (i.e., emotional avoidance or intolerance). It is important to note that observed EDE-Q effect sizes were already quite large in treatment-as-usual, and may have demonstrated ceiling effects. Additional analyses are required, however, to ascertain what additional interventions could provide relative benefit for patients whose symptoms are severe and intractable, but do not have emotion avoidance.

Analyses of depression outcome, as well as experiential avoidance, anxiety sensitivity, and mindfulness, all indicated that individuals treated in the UT phase showed greater improvements relative to patients treated prior to the implementation in the TAU phase. In the model of anxiety sensitivity, there was also a significant moderating effect of baseline experiential avoidance, suggesting that this relatively larger benefit of the UT on ASI score was more pronounced among those individuals who had higher BEAQ admission scores. This finding again supports the supposition that an emotion-focused transdiagnostic intervention, such as the UT, addresses the co-occurring emotion dysfunction that is characteristic of a large proportion of patients who enter residential treatment.

Findings regarding the effects of the treatment for patients specifically with AN-R were unexpected and require additional investigation. Patients who entered treatment with an AN-R diagnosis reported lesser decrease in eating disorder symptoms, depression, experiential avoidance, and mindfulness from intake to discharge; however, AN-R was associated with *greater* change in anxiety sensitivity from intake to discharge. These findings concerned the subsample with AN-R relative to all other patients, and were not specific to treatment type. This pattern is not easy to explain, and requires deeper analysis of baseline differences between diagnostic groups, the relationship between change in these variables and change in weight during residential treatment for individuals with AN, and the general question of treatment-resistance. Individuals with AN-R are observed to have reduced awareness of emotions, though comparisons between individuals with active AN-R and recovered individuals, individuals with AN-binge/purge type, and individuals with other ED diagnoses are complex and require additional study (67). This study did not investigate body mass index or ideal body weight as an outcome, in part due to the limitations of our self-reported follow-up measurements. Further research is needed to investigate these important questions.

Visual inspection of treatment effect sizes for all variables calculated by year indicated that the effects observed in the immediate full implementation period were largely sustained across subsequent years. Although descriptive, the observed trends reflect consistency over time. This is notable given the complexity of residential care and the routine variability in staff over time in such settings. Previous implementation work has warned that “drift” overtime is commonplace (56); at the very least, the pattern of effect sizes indicates the absence of a worsening trend over a 5 year period.

It is important to keep in mind that treatment only occurred between intake and discharge and, in general, it was expected that the larger the observed effect was at the time of discharge, some degree of rebound would occur between discharge and follow-up. Some degree of relapse is common post-discharge (67), as well as the likelihood of regression to the mean. Nonetheless, in many cases there was still a significant benefit to the UT at 6MFU, particularly for experiential avoidance, anxiety sensitivity, and mindfulness. These reflect aspects of emotional functioning that were directly addressed in the UT, as well as unique improvements in ED symptoms for those who entered treatment with a high level of experiential avoidance.

This study had several limitations. As a community case example that included a wide-scale EBP implementation across a complex system of care, there were many changes associated with the implementation as well as the passage of time that were not directly measured or isolated. As such, we cannot determine which specific elements of sustainment effort are associated with maintenance of treatment fidelity or patient outcomes. Because patients were not randomized or treated in concurrent time periods, it is possible that cohort differences or other changes in treatment associated with the passage of time accounted for observed differences. We tried to include covariates that reflected any variable that did seem to differ across time periods, but many potential covariates were not measured. Notably, response rates differed over time; as research procedures in the programs improved over time, the response rate went up. We examined whether there were systematic differences associated with response rate, and importantly, no differences on any outcome measures at admission were observed between completers and non-completers.

The study's limitations regarding the race and gender of the participants deserve additional comment. The underrepresentation of non-Caucasian participants in the sample reflects the underrepresentation of people of color across mental health treatment and research. The elimination of these disparities must be a high priority for providers, policy makers, and researchers. These problems may be exacerbated in residential/inpatient treatment programs, which are often private and costly, and therefore engage issues of intersectionality and structural racism. Furthermore, residential treatment programs are *de facto* communities, where racial/ethnic and gender minorities may feel the effects of discrimination or marginalization in unique ways. The Renfrew Treatment Center has undertaken research to understand the experience of patients who identify as minorities in terms of their race/ethnicity, gender, or sexuality, and we are committed to making substantial effects to identify and rectify any problems at every level.

Despite these limitations, the substantive data collected across multiple years of treatment suggest that the implementation of an EBP at two residential treatment programs was associated with stronger effects in intended outcome areas, in some cases particularly for patients who had higher levels of emotional intolerance, which the EBP was intended to address. These complex patients typically have co-occurring emotional disorders and are regularly treated in higher levels of care such as residential treatment. The results from this study suggest that it is possible to implement an integrative EBP protocol to address both eating disorder and emotion functioning symptoms, and to sustain that effect over multiple years.

## Data Availability Statement

The raw data supporting the conclusions of this article will be made available by the authors, without undue reservation.

## Ethics Statement

The studies involving human participants were reviewed and approved by Drexel University. Written informed consent to participate in this study was provided by the participants' legal guardian/next of kin.

## Author Contributions

All authors listed have made a substantial, direct and intellectual contribution to the work, and approved it for publication.

## Conflict of Interest

The authors include Research Consultants to The Renfrew Center (HT-B, ML, SS); Advisory Board Members for The Renfrew Center (JB), and employees of The Renfrew Center (TG, GB, MS). The remaining author declares that the research was conducted in the absence of any commercial or financial relationships that could be construed as a potential conflict of interest.

## Footnotes

^1^Note that the direction of the Southampton Mindfulness Questionnaire is different from the other outcome measures, and improvement is indicated by higher scores/increases rather than lower scores/decreases.
